# Clinical and Microbiological Evaluation of Local Doxycycline and Antimicrobial Photodynamic Therapy during Supportive Periodontal Therapy: A Randomized Clinical Trial

**DOI:** 10.3390/antibiotics10030277

**Published:** 2021-03-09

**Authors:** Raluca Cosgarea, Sigrun Eick, Ionela Batori-Andronescu, Søren Jepsen, Nicole B. Arweiler, Ralf Rößler, Torsten Conrad, Christoph A. Ramseier, Anton Sculean

**Affiliations:** 1 Department for Periodontology, Operative and Preventive Dentistry, University of Bonn, 53111 Bonn, Germany; sjepsen@uni-bonn.de; 2 Clinic for Periodontology and Peri-Implant Diseases, Philipps University Marburg, 35033 Marburg, Germany; arweiler@med.uni-marburg.de; 3 Department of Prosthodontics, Iuliu Hatieganu University Cluj-Napoca, 400006 Cluj-Napoca, Romania; 4 Department of Periodontology, School of Dentistry, University of Bern, 3010 Bern, Switzerland; sigrun.eick@zmk.unibe.ch (S.E.); christoph.ramseier@zmk.unibe.ch (C.A.R.); anton.sculean@zmk.unibe.ch (A.S.); 5 Department, Periodontal Private Practice Cosmedica, 400185 Cluj-Napoca, Romania; andronescu.ionela@gmail.com; 6 University of Digital Technologies in Medicine & Dentistry, 9516 Wiltz, Luxembourg; ralf.roessler@dtmd.eu (R.R.); Torsten.Conrad@dr-conrad.de (T.C.); 7 Clinic for Mouth, Jaw and Plastic Facesurgery, University of Frankfurt, 60590 Frankfurt, Germany; 8 Private Practice, 55411 Bingen am Rhein, Germany

**Keywords:** supportive periodontal therapy, photodynamic therapy, local drug delivery, periodontal treatment, persistent periodontal pockets

## Abstract

The aim of this study was to evaluate the clinical and microbiological effects of subgingival instrumentation (SI) alone or combined with either local drug delivery (LDD) or photodynamic therapy (PDT) in persistent/recurrent pockets in patients enrolled in supportive periodontal therapy (SPT). A total of 105 patients enrolled in SPT were randomly treated as follows: group A (*n* = 35): SI +PDT and 7 days later 2nd PDT; group B (*n* = 35): SI+LDD; group C (*n* = 35): SI (control). Prior intervention, at 3 and 6 months after therapy, probing pocket depths, clinical attachment level, number of treated sites with bleeding on probing (n BOP), full mouth plaque and bleeding scores (gingival bleeding index, %BOP) were recorded. At the same time points, 8 periodontopathogens were quantitatively determined. All three treatments resulted in statistically significant improvements (*p* < 0.05) of all clinical parameters without statistically significant intergroup differences (*p* > 0.05). Several bacterial species were reduced in both test groups, with statistically significantly higher reductions for LDD compared to PDT and the control group. In conclusion, the present data indicate that: (a) In periodontal patients enrolled in SPT, treatment of persistent/recurrent pockets with SI alone or combined with either PDT or LDD may lead to comparable clinical improvements and (b) the adjunctive use of LDD appears to provide better microbiological improvements for some periodontal pathogens than SI alone or combined with PDT.

## 1. Introduction

Supportive/maintenance periodontal therapy (SPT) aims at preventing or at least minimizing the recurrence of disease and its progression in previously treated periodontitis patients and is currently referred to as step 4 of therapy in the EFP S3 (European Federation of Periodontology) clinical practice guideline [1,2]. SPT consists of predefined recall sessions, with re-examination of the periodontal status, re-instrumentation of sites with clinical signs of disease activity, re-enforcement of the self-performed oral hygiene, and polishing of the teeth. Sites with disease activity are considered those with probing depths (PD) ≥4 mm and bleeding on probing (BOP) [2,3,4]. The adjunctive use of antimicrobials or of different types of lasers, especially the photodynamic therapy (PDT), has been investigated for the treatment of recurrent sites in SPT patients [5,6,7,8,9].

The use of local antimicrobials in SPT patients has been investigated in numerous controlled clinical studies and systematic reviews, reporting on greater clinical improvements in terms of PD reduction and clinical attachment level (CAL) gain for the adjunctive use of locally applied antimicrobials to subgingival instrumentation (SI) as compared to SI alone [10]. Moreover, subgingival application of a 14% doxycycline local drug delivery (LDD) in residual/recurrent deep sites showed comparable results to SI [11]. Positive short-term effects of topically applied doxycycline were also reported in furcation-involved teeth in SPT [8].

PDT has also been investigated for possible adjunctive improvements for periodontal treatment. PDT has been used for medical purposes since 1904. The therapy is based on the light-induced inactivation of bacterial cells: a photosensitizer (e.g., toluidine blue or methylene blue) that absorbs light, binds to target cells. Exposure to light in a suitable wavelength in the presence of molecular oxygen generates singlet oxygen and free radicals that are cytotoxic to microorganisms [12,13,14,15,16]. The photosensitizer toluidine blue in combination with a helium/neon soft laser irradiation was highly efficient regarding the in vitro elimination of periodontopathogens [16,17,18,19]. The clinical efficiency of PDT as an adjunct to SI was investigated both for the initial “cause-related” [20,21] as well as for the maintenance therapy [6,7,9], partly showing promising clinical, microbiological, and immunological improvements. However, controlled clinical trials (RCTs) comparing the effects of PDT used in conjunction to SI or as an alternative, reported conflicting results with regard to the pathogen elimination and clinical improvement both in active as well as in SPT [7,9,22,23,24,25,26]. The great advantage of PDT is the lack of an increase in bacterial resistance, which automatically points to the clinical relevance of this treatment approach in the treatment of chronic biofilm-associated diseases such as periodontitis or peri-implantitis. The absence of genotoxic and mutagenic effects of PDT is an important factor for long-term safety during treatment and thus, PDT represents a clinically relevant therapeutic approach in the management of oral biofilms [27,28].

Only a few studies have compared the efficacy of PDT and LDD. Some authors showed no significant difference between PDT or minocycline microspheres applied adjunctively to SI in untreated periodontitis [29]; similar findings were also reported for peri-implantitis lesions, where the results indicated that the adjunctive application of PDT was equally effective in the reduction of mucosal inflammation as the adjunctive delivery of minocycline microspheres up to 12 months [30].

Since the repeated use of antibiotics is the major cause for the increase in antibiotic resistance worldwide, and considering that the repeated topical application of antibiotics may also contribute to antibiotic resistance, it seems relevant to further explore the effects of PDT as an alternative to adjunctive antibiotics in periodontal treatment.

However, at present, very limited information is available on the potential effects of PDT as compared with that of locally delivered doxycycline (LDD) when used adjunctive to SI in SPT patients. Therefore, the aim of this study was to evaluate the clinical and microbiological effects of subgingival instrumentation (SI) alone or combined with either photodynamic therapy (PDT) or antibiotic local drug delivery (LDD) in persistent/recurrent pockets of periodontal patients enrolled in supportive periodontal therapy (SPT).

## 2. Results

**Figure 1:**
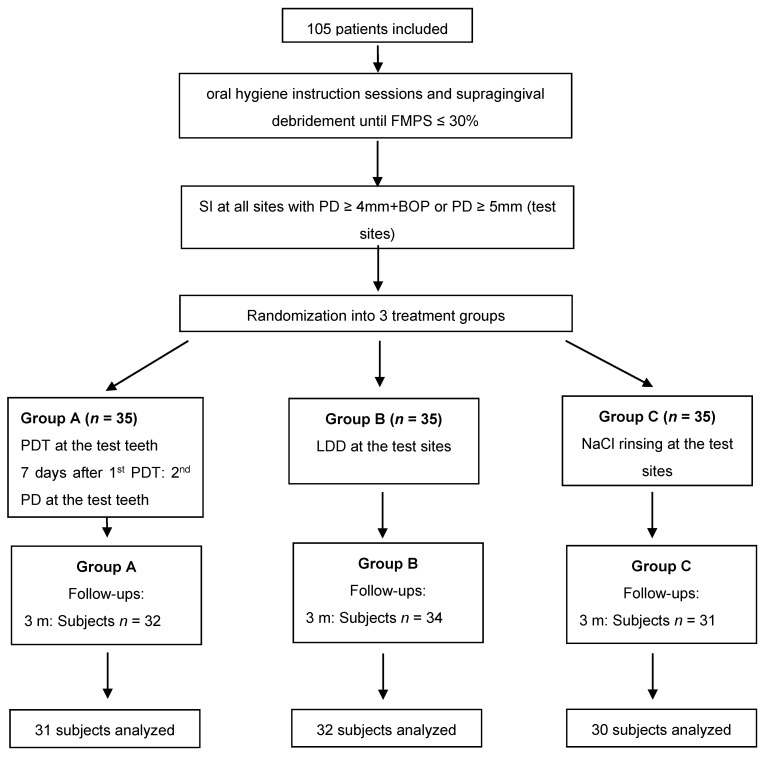
Flowchart of the study (FMPS: full mouth plaque score; SI: subgingival instrumentation; LDD: local drug delivery; PDT: photodynamic therapy).

A total of 105 patients diagnosed with stages I–IV, grades A, B, C periodontitis (*n* = 35 per treatment group) were included in the present study. Of those, 93 subjects completed the 6 months evaluation (Figure 1). Baseline demographics including mean age, disease severity, and smoking history are summarized in Table 1. No statistically significant differences between the groups could be found related to age or gender. Nonetheless, statistically significantly more smokers were found in the antibiotics and control group as compared to the laser group. The majority of the patients were diagnosed with stage III grade B periodontitis (Table 1).

Table 2 depicts the full mouth clinical data for BOP, plaque scores, and gingival bleeding index. No statistically significant differences could be recorded between the groups at any of the evaluated timepoints. Intragroup comparisons revealed statistically significant BOP reductions in the laser group. Full mouth plaque score (FMPS) showed an increase within 6 months in all groups; however, a statistically significant FMPS increase was noticed only in the LDD and control groups.

BOP at test teeth showed statistically significant reductions from baseline up to 6 months in all treatment groups, with no statistically significant intergroup differences (*p* > 0.05, Table 3).

Similarly, statistically significant PD reductions were observed in all three groups, without any statistical intergroup differences at any of the evaluated timepoints (Table 4). CAL values on the other hand, differed statistically significantly between the groups at baseline, while no differences could be observed at the follow-ups (Table 5). Nonetheless, CAL was statistically significantly improved at 3 and at 6 months in all three groups.

Microbiological analyses revealed in the laser group no change of the investigated periodontal pathogens. In the antibiotic group, there was a decrease of *Treponema denticola, Tannerella forsythia,* and *Filifactor allocis*, while the control group counts of *T. denticola* and *Fusobacterium nucleatum* increased (Table 6).

Group comparisons indicated differences at 3 and 6 months for *T. denticola, T. forsythia, P. intermedia,* and *F. alocis* with the lowest counts always in the antibiotics group (Table 6).

## 3. Discussion

This prospective randomized clinical trial has evaluated the clinical and microbiological effects of SI alone or combined with either PDT or LDD in persistent/recurrent pockets of patients enrolled in SPT. The treatment was performed only at baseline and all clinical and microbiological parameters were re-evaluated at 3 and 6 months after therapy.

The test site-specific outcomes indicated statistically significant improvements in all three treatment groups and for all investigated clinical parameters. For the main outcome variable, the number of bleeding sites at 6 months, no statistically significant group differences could be found. Interestingly, at 3 months, BOP reductions in all groups were much smaller as compared to those obtained at 6 months. Similar outcomes were also found for PD reduction. On the other hand, CAL, even though statistically insignificant, showed the greatest improvements in the test groups as compared to the control group. Even though at baseline, statistically significant differences were found between the treatment groups (Kruskal–Wallis test), after Bonferroni adjustments, no statistically significant differences were detected between the groups.

Comparable clinical outcomes in terms of PD reduction and CAL gain to those obtained in the PDT group, were also reported by other authors evaluating the efficacy of this treatment modality in patients enrolled in SPT [7,31,32,33,34,35]. Even though not all these studies had comparable initial clinical parameters (higher initial PD values) [7,31,32,34,35] with those in our study, and used slightly different treatment protocols (i.e., scaling and root planing with hand curettes [9] as opposed to SD with ultrasonics, repeated PDT sessions more than twice [31,34,36], or no additional mechanical biofilm removal in the PDT group [32]), the changes in PD or CAL were reaching up to 0.8 mm, similar to our results. The differences between the baseline PD values of these authors and our study rely on the fact that our reported clinical values represent the mean values of all 6 sites of the test teeth, which also include sites with PD < 4 mm. Interestingly, BOP changes in our study were much smaller as compared to those reported by other authors [6,7,9,31,35,37]. This may be due to the increase in full mouth plaque scores at both follow-ups in our study, as opposed to the other authors that reported a decrease in plaque scores [6,7,9,31,35,37]. Additionally, discrepancies in treatment protocols may also explain the various clinical results: Chondros et al. excluded from the analyses sites with clinical deterioration during the experimental period [7]; Kolbe et al. used for subgingival instrumentation both curettes and ultrasonic and utilized a different type of laser [9]; and Lulic et al. and Petelin et al. used PDT more than twice [31,34]. Nonetheless, despite these discrepancies for the reported PD, CAL, and BOP values, several of these authors reported no statistically significant adjunctive benefits for the PDT as compared to mechanical debridement [9,32,33,35,37] which is in line with our results. Other authors reported significant differences favoring PDT only for the parameter BOP, without any significant benefits for PD or CAL [7,31], while other authors reported significant improvements in all parameters favoring PDT treatment [6,36].

Due to limited availability and difficulty in preparation of locally delivered antibiotics in various countries worldwide, only few studies evaluated their efficacy during supportive periodontal therapy [5,8,11,38,39,40]. Comparing the present results of the subjects receiving LDD with those in other studies, it appears that lower baseline as well as follow-up changes for PD and CAL were recorded in the present study compared to other authors [5,8,39,40,41]. This relies on the fact that in the present paper, as previously mentioned, mean values of the test teeth were considered for statistical analyses, since SD was performed at all sites of the test-tooth. Nonetheless, in all studies including the present one, statistically significant clinical improvements (BOP, PD, CAL) compared to baseline were obtained, and intergroup comparisons revealed no statistically significant additional benefits for follow-ups at 6 months or later compared to mechanical debridement alone [5,8,39,40,41]. However, short-term statistically significant improvements favoring LDD were reported by some authors at 3 months [5,8,40]. Corroborating these data, the clinical improvements in our study were slightly higher at 3 months compared to those at 6 months, confirming the suggestion of previous reports on the limited time-dependent (short-time) effects of adjunctive topical administration of doxycycline [5,40].

Microbiologically, two of the eight investigated periodontal pathogens in the control group showed a quantitative increase at both follow-ups. Additionally, *Porphyromonas gingivalis* and several other bacteria did not show statistically significant reductions in any of the three groups. This may be explained by the slight increase in full mouth plaque scores in all three groups which have a substantial impact on the level of anaerobic subgingival bacteria. Similarly, this may also explain the contradiction of these findings compared to the reported microbiological improvements in the PDT treated subjects of other studies [7,9,31,37]. However, similar to our results, Rühling et al. and Müller-Campanile et al. could not find any statistically significant bacterial improvements either [32,35]. Considering intergroup comparisons, some of the investigated bacteria were present in statistically significantly lower counts in the LDD group compared to PDT (*p* < 0.05, *T. denticola*, *T. forsythia*, *P. intermedia*, and *F. allocis*), as well as compared to the control group (*p* < 0.05, *P. gingivalis*, *T. denticola*, *T. forsythia*, and *F. allocis*). Corroborating the present data, statistically significantly lower bacterial counts of the red complex were reported in subjects receiving LDD compared to those in the control group after 2 years by Bogren et al. [5]. The finding that not many of the investigated pathogens were statistically significantly reduced in groups A and C, despite the obtained statistically significant clinical improvements, may be explained by the clinical effect obtained by removing the subgingival biofilm. Additionally, no statistically significant changes of the subgingival flora may be expected in such a short time despite the slight increase in the amount of supra-gingival biofilm [42].

Considering the fact that some authors reported improved clinical outcomes when PDT was repeatedly used [30,31,34,36,43], we applied PDT seven days after the initial PDT again.

The reason for choosing BOP as primary outcome variable was based on previous findings indicating that the major effects following the use of PDT in patients enrolled in SPT are the improvements in terms of BOP and not necessarily the changes in PD and CAL [7,30,31,43]. Additionally, BOP is a commonly accepted clinical sign of inflammation which, combined with a PD ≥ 4 mm, has been shown to represent a potential risk for further attachment loss [4]. The rationale to include LDD as second test groups was based on the limited studies comparing its use with that of PDT. Only one RCT for step 2 periodontal therapy (anti-infective periodontal treatment) compared the clinical and microbiological efficacy of PDT and LDD (minocycline microspheres) adjunctive to SI indicating improvements in all treatment groups with no statistically significant difference between the treatments [29]. However, no study has evaluated so far the adjunctive benefits of LDD (doxycycline) or PDT over SI alone for step 4 periodontal therapy.

Limitations of the present study include the lack of stratification in the randomization procedures regarding smoking and all baseline clinical parameters. Nonetheless, despite the general statistically significant difference for baseline CAL values, no statistically significant differences between the groups were found after Bonferroni corrections. Additionally, the inclusion of smokers neither influenced their group distributions nor the clinical results, since a separate analysis between smokers and non-smokers revealed no statistically significant differences for the clinical outcomes.

## 4. Material and Methods

### 4.1. Study Protocol and Participants

This was a prospective, randomized, single-blinded clinical trial. The study was conducted according to the Declaration of Helsinki (1964, revision 2008) and approved by the Ethical Committee of the Faculty of Medicine and Pharmacy of Cluj-Napoca (Application #390/02.07.2015). The study has been registered in the ISRCTN trial registry (registration ID: ISRCTN17209965, https://www.isrctn.com/ISRCTN17209965, accessed on 26 February 2021). All included subjects signed an informed consent prior to participation in the study.

A total of 105 patients with stages I–IV, grade A/B/C periodontitis (previously chronic periodontitis) in SPT with at least four teeth, each with a minimum of one site with PD ≥ 4 mm and BOP+ or PD ≥ 5 mm, who were in SPT, were enrolled and treated in this study. In order to be included, patients had to be enrolled in SPT (minimum 6 months after completion of active periodontal therapy), a minimal age of 35 years, minimum four sites at different teeth with PD ≥ 4 mm and BOP+ or PD ≥ 5 mm, good level of oral hygiene (plaque control record (FMPS) after O’Leary 1972 ≤ 30%) [44], and to be systemically healthy: no history of diseases that may influence the severity or progression of the periodontal disease (Down syndrome, HIV, diabetes mellitus type 1 and 2), post-irradiation in the head and neck area, infectious diseases or heart diseases that need a prophylactic antibiosis before dental treatments, or liver diseases.

Patients smoking over 10 cigarettes/day, or taking systemic or local antibiotics within the preceding six months, or medication that may have interacted with Doxycycline (e.g., coumarin derivates, containing alcohol derivates, 5-fluor-uracyl/disulfiram derivates, amprenavir oral solutions, lopinavir/ritonavir oral solution) or may have influenced the periodontium (Ciclosporin A, compounds of Phenytoin, calcium channel blockers), or pregnant patients were excluded from participation in the study.

### 4.2. Clinical Protocol

According to a computer-generated randomization list, patients were assigned to one of the following treatment groups (Flowchart of the study Figure 1):

Group A: session 1: SI plus PDT
  session 2: PDT (7 days later)
Group B: session 1: SI plus LDD
Group C: session 1: SI alone

The randomization list was concealed from the patient, clinical examiner, therapist, and statistician. SI was performed using ultrasonic instruments (EMS, Piezon Master 700, Switzerland) at all teeth (test teeth) with sites with PD ≥ 4 mm and BOP+ or PD ≥ 5mm. Thereafter, patients were treated according to the randomization code:

**Group A:**

Session 1: Five minutes after SI and ceasing of bleeding, the photosensitizer (HELBO Blue Photosensitizer, Bredent medical, Senden, Germany) was applied at 6 sites per test tooth from the bottom to the top of the pocket and left in situ for 3 min. Subsequently, the dye (phenothiazine chloride) was rinsed off with sterile saline solution and each site of the treated tooth was exposed to laser light for 10 s (HELBO TheraLite Laser, HELBO 3D Pocket Probe, Bredent medical, Senden, Germany) at a wavelength of 660 nm and an output power of 100 mW.
Session 2: after 1 week, PDT was repeated at all test teeth as previously described.

**Group B:** At all treated sites, LDD (Ligosan, Heraeus Kulzer, Germany) was applied to the bottom of the pocket. All subjects avoided interdental flossing/brushing for the following 10 days to avoid the mechanical removal of the LDD.

**Group C:** All treated sites were rinsed with sterile saline solution.

Subjects in groups A and C were instructed to perform their usual oral hygiene procedures.

All treatments were performed by one experienced periodontist (R.C.) and all clinical examinations were performed by one blinded and calibrated periodontist (I.A.). The examiner was calibrated by measuring PD and CAL in five patients two times, 48 h apart. Positive calibration was considered when both measurements were within one millimeter more than 90% of the times.

At baseline (before therapy), at 3 months, and at 6 months after therapy, medical history, smoking history (patients smoking >10 cigarettes per day will be considered smokers [45], patients who stopped smoking 5 years before the beginning of the study will be considered former smokers), and periodontal status were recorded. Periodontal recording included assessment of PD, clinical vertical attachment levels (CAL) at 6 sites per tooth with a periodontal probe (PCPUNC 15; Hu Friedy^®^, Chicago, IL, USA) to the nearest mm. As a reference point for CAL measurements, the cement-enamel junction (CEJ) was used. If the CEJ was covered by a restoration (filling or crown), the margin of the restoration was considered as a reference point. Furthermore, bleeding on probing (BOP), suppuration on probing (SOP), gingival bleeding (GBI according to Ainamo and Bay 1975) [46], and full mouth plaque score (FMPS according to O’Leary 1972) [44] were recorded. Additionally, microbial samples were obtained from the four deepest sites, using sterile paper points. All samples were stored at −20° until microbiological analysis for determining the periodontal pathogens *Aggregatibacter actinomycetemcomitans*, *Porphyromonas gingivalis*, *Tannerella forsythia*, *Prevotella intermedia*, *Treponema denticola*, *Fusobacterium nucleatum*, *Campylobacter rectus*, and *Filifactor allocis* by real-time PCR [47].

### 4.3. Statistical Analysis

Sample size calculation was performed considering the possibility to detect a difference of 1 bleeding site (BOP positive) out of 6 sites per tooth with a standard deviation of 1.3 [30,48]. A study power of ≥85% at a statistical significance level of 0.05 was to be reached by including 30 subjects per treatment group. The statistical unit was the patient while the primary outcome variable was the number of sites with BOP. Secondary variables were means and changes in PD, CAL, and levels of *A. actinomycetemcomitans*, *P. gingivalis*, *T. forsythia*, *P. intermedia, T. denticola*, *F. nucleatum*, *C. rectus*, and *F. allocis.* Statistical analyses were performed using SPSS and RStudio (Version 1.3.1093, RStudio Team (2020). RStudio: Integrated Development Environment for R. RStudio, PBC, Boston, MA URL http://www.rstudio.com/, accessed on 8 March 2021). Mean, percentage, and standard deviation values were calculated by means of descriptive statistics. Chi-square tests, Mann–Whitney tests, Student’s *t*-test, Kruskal–Wallis tests, Friedman tests, and one-way analysis of variance (ANOVA) were used to test for statistical significance of differences between numerical variables within subgroups. Post hoc analyses were performed and consecutive comparisons of follow-up data were adjusted using Bonferroni tests. *p* values < 0.05 were defined as statistically significant. Microbial data were analyzed using the statistical software program SPSS (IBM Corp. Released 2019. IBM SPSS Statistics for Windows, Version 26.0. Armonk, NY: IBM Corp). Intragroup comparisons of mean bacterial counts were analyzed by means of Friedman test and intergroup differences using the Kruskal–Wallis test.

## 5. Conclusions

In conclusion, within their limits the present data indicate that: (a) In periodontal patients enrolled in SPT treatment of persistent/recurrent pockets with SI alone or combined with either PDT or LDD may lead to comparable clinical improvements and (b) the adjunctive use of LDD appears to provide better microbiological improvements for some periodontal pathogens than SI alone or combined with PDT.

## Figures and Tables

**Table 1:** Demographics at baseline of *n* = 105 patients randomized into three groups (A: PDT, B: LDDs, C: control) of *n* = 35 patients each.

Parameters	All			Group A			Group B			Group C			Group Comparisons
	n	%	Mean ± SD	n	%	Mean ± SD	n	%	Mean ± SD	n	%	Mean ± SD	*p*-Value
n	105	100		35	33.33		35	33.33		35	33.33		
Gender (female/male)	64/41	61/39		21	60.00		23	65.71		20	57.14		0.756 (¶)
Age			46 ± 10			46 ± 11			48 ± 9			44 ± 9.15	0.480 (§)
Diagnosis													
Stage I grade A	3	2.86		1	2.86		0	0.00		2	5.71		
Stage II grade A	1	0.95		0	0.00		1	2.86		0	0.00		
Stage II grade B	16	15.24		6	17.14		1	2.86		9	25.71		
Stage III grade A	2	1.90		1	2.86		0	0.00		1	2.86		
Stage III grade B	44	41.90		17	48.57		15	42.86		12	34.29		
Stage III grade C	19	18.10		5	14.29		6	17.14		8	22.86		
Stage IV grade B	10	9.52		3	8.57		6	17.14		1	2.86		
Stage IV grade C	10	9.52		2	5.71		6	17.14		2	5.71		
Non-smokers	76	72.38		30	85.71		23	65.71		23	65.71		
Smokers	23	21.90		2	5.71		9	25.71		12	34.29		0.018 (£)
Former smokers	6	5.71		3	8.57		3	8.57		0	0.00		

(¶) Chi-Square-Test, X^2^ = 0.5602. (§) Analysis of Variance (ANOVA). (£) Chi-Square-Test, X^2^ = 8.0017. SD standard deviation.

**Table 2:** Full mouth scores: bleeding on probing (BOP, %), gingival bleeding score (GBI, %), and plaque score (FMPS, %).

Parameters	All				Group A				Group B				Group C				Inter-Group
	Mean	SD	min	max	Mean	SD	min	max	Mean	SD	min	max	Mean	SD	min	max	*p*-Value (£)
BOP (BL)	16.82	8.67	3.33	42.3	16.52	8.31	4.16	42.3	17.36	9.33	4.2	41.1	16.57	8.57	3.33	39.8	0.949
BOP (3 MO)	13.37	7.54	1.6	42.7	12.29	6.1	2.5	23.9	13.9	8.17	1.6	42.7	13.92	8.28	2.4	38.7	0.779
BOP (6 MO)	12.66	7.6	0	41.6	10.75	7.12	0	26.6	13.23	9.11	0	41.6	14.04	6.07	1.8	25.9	0.133
*p*-value (*)	0.003				**0.038**				**0.113**				**0.312**				
GBI (BL)	3.67	6.07	0	28	2.56	4.5	0	22.4	4.39	6.75	0	27.7	4.07	6.71	0	28	0.829
GBI (3 MO)	4.77	5.61	0	21.3	3.4	4.41	0	15.4	5.37	6.02	0	21.3	5.54	6.17	0	17.8	0.237
GBI (6 MO)	4.59	5.99	0	22.8	3.85	5.54	0	22.8	4.92	5.65	0	19	5	6.86	0	22.3	0.713
*p*-value (*)	0.007				**0.282**				**0.094**				**0.227**				
FMPS (BL)	18.43	6.97	1.7	33	18.32	7.32	4.2	27.3	18.39	6.2	7	33	18.57	7.51	1.7	29	0.736
FMPS (3 MO)	22.69	12.34	0	59.7	21.51	11.76	4.3	59.7	24.94	12.48	3.8	52.7	21.4	12.84	0	50.8	0.401
FMPS (6 MO)	24.71	14.64	0	66	21.88	10.9	2.5	53.8	24.53	14.22	0	64.4	27.83	17.94	0	66	0.472
*p*-value (*)	0.002				**0.407**				**0.038**				**0.0223**				

(£) Kruskal-Wallis-Test. (*) Friedman-Test. BOP: bleeding on probing; GBI: gingival bleeding index; FMPS: full-mouth plaque score; MO: months.

**Table 3:** Mean number of BOP+ sites ± standard deviation (SD) per tooth (six-point measurement) in the three treatment groups (A: PDT, B: LDDs, C: control) at baseline and after 3 and 6 months.

Groups	Baseline					3 Months					6 Months					Intra-Group	
	n pat.	Mean n Teeth (±SD)	Mean BoP+ Sites/Tooth (±SD)	min	max	n pat.	Mean n Teeth (±SD)	Mean BoP+ Sites/Tooth (±SD)	min	max	n pat.	Mean n Teeth (±SD)	Mean BoP+ Sites/Tooth (±SD)	min	max	*p*-Value	
Group A	35	5.43 (±1.96)	1.71 (±0.69)	0.38	3.20	32	4.91 (±2.43)	1.21 (±0.75)	0.00	3.50	31	4.89 (±2.62)	0.17 (±0.12)	0.00	0.52	<0.001	(*)
Group B	35	5.34 (±2.13)	1.82 (±0.78)	0.50	3.60	34	4.97 (±1.87)	1.12 (±0.68)	0.25	3.00	32	4.97 (±2.61)	0.16 (±0.12)	0.00	0.43	<0.001	(*)
Group C	35	5.49 (±2.17)	1.92 (±0.91)	0.60	4.25	31	5.00 (±2.80)	1.12 (±0.70)	0.00	3.50	30	5.11 (±3.35)	0.20 (±0.11)	0.04	0.47	<0.001	(*)
***p*-value (intergroup)**			**0.778**	**(£)**				**0.835**	**(£)**				**0.182**	**(£)**			

(£) Kruskal-Wallis-Test. (*) Friedman-Test; n pat.: number of patients

**Table 4:** Pocket probing depth (mm) ± SD per tooth in the three treatment groups (A: PDT, B: LDDs, C: control) at baseline and after 3 and 6 months.

Groups	Baseline					3 Months					6 Months					Intra-Group	
	n pat.	Mean n Teeth (±SD)	Mean PD (±SD)	min	max	n pat.	Mean n Teeth (±SD)	Mean PD (±SD)	min	max	n pat.	Mean n Teeth (±SD)	Mean PD (±SD)	min	max	*p*-Value	
Group A	35	5.43 (±1.96)	2.96 (±0.30)	2.37	3.67	32	4.91 (±2.43)	2.72 (±0.30)	2.23	3.63	31	4.89 (±2.62)	2.75 (±0.39)	2.06	3.46	0.001	(*)
Group B	35	5.34 (±2.13)	2.94 (±0.20)	2.50	3.38	34	4.97 (±1.87)	2.64 (±0.26)	2.17	3.33	32	4.97 (±2.61)	2.66 (±0.28)	2.11	3.30	<0.001	(*)
Group C	35	5.49 (±2.17)	2.97 (±0.24)	2.63	3.54	31	5.00 (±2.80)	2.66 (±0.30)	2.21	3.49	30	5.11 (±3.35)	2.71 (±0.34)	2.17	3.80	<0.001	(*)
***p*-value (intergroup)**			**0.969**	**(£)**					**0.453**	**(£)**					**0.641**	**(£)**	

(£) Kruskal-Wallis-Test. (*) Friedman-Test. PD: probing pocket depth; n pat.: number of patients.

**Table 5:** Clinical attachment level (mm) ± SD per tooth in the three treatment groups (A: PDT, B: LDD, C: control) at baseline and after 3 and 6 months).

Groups	Baseline					3 Months					6 Months					Intra-Group	
	n pat.	Mean n Teeth (±SD)	Mean CAL (±SD)	min	max	n pat.	Mean n Teeth (±SD)	Mean CAL (±SD)	min	max	n pat.	Mean n Teeth (±SD)	Mean CAL (±SD)	min	max	*p*-Value	
Group A	35	5.43 (±1.96)	3.67 (±0.81)	2.33	5.82	32	4.91 (±2.43)	3.48 (±0.90)	2.08	5.99	31	4.89 (±2.62)	3.53 (±0.86)	1.96	5.99	0.004	(*)
Group B	35	5.34 (±2.13)	4.13 (±0.97)	2.88	7.00	34	4.97 (±1.87)	3.91 (±1.12)	2.54	7.00	32	4.97 (±2.61)	3.83 (±1.10)	2.42	7.25	<0.001	(*)
Group C	35	5.49 (±2.17)	3.66 (±0.83)	2.39	6.15	31	5.00 (±2.80)	3.38 (±0.84)	2.31	6.12	30	5.11 (±3.35)	3.51 (±0.88)	2.19	5.58	<0.001	(*)
***p*-value (intergroup)**			**0.041**	**(£)**				**0.064**	**(£)**					**0.409**			

(£) Kruskal-Wallis-Test. (*) Friedman-Test. CAL: clinical attachment level; n pat.: number of patients.

**Table 6:** Mean counts (±SD) of eight periodontal pathogens in the three treatment groups (A: PDT, B: LDD, C: control) at baseline and after 3 and 6 months.

Variables	Group A	Group B	Group C	Inter-Group *p*-Value (£)
***A. actinomycetemecomitans* (log10)**				
Baseline	0.74 ± 1.67	0.68 ± 1.72	0.63 ± 1.79	0.899
3 m	0.51 ± 1.37	0.70 ± 1.59	0.47 ± 1.47	0.713
6 m	0.31 ± 1.15	0.70 ± 1.58	0.20 ± 1.07	0.235
*p* value (*)	**0.094**	**0.580**	**0.905**	
***P. gingivalis* (log10)**				
Baseline	3.45 ± 2.97	3.69 ± 2.97	3.84 ± 2.83	0.919
3 months	3.66 ± 3.12	2.92 ± 2.72 ^a^	3.85 ± 2.96	0.201
6 months	3.74 ± 2.96	2.64 ± 3.04	4.26 ± 3.11 ^c^	0.103
*p* value (*)	**0.314**	**0.170**	**0.158**	
***T. denticola* (log10)**				
Baseline	3.78 ± 2.96	3.06 ± 2.90	3.63 ± 2.50	0.611
3 m	3.78 ± 2.80	2.13 ± 2.51 ^a^	3.63 ± 2.60 ^a^	**0.013 ^s^**
6 m	3.66 ± 2.78 ^c^	2.29 ± 2.65	4.11 ± 2.55 ^a,b^	**0.015 ^s^**
*p* value (*)	**0.291**	**0.014**	**0.026**	
***T. forsythia* (log10)**				
Baseline	4.88 ± 2.36	4.72 ± 2.53	4.61 ± 2.68	1.000
3 m	5.30 ± 2.02	4.04 ± 2.63 ^a^	4.93 ± 2.31	**0.034**
6 m	5.04 ± 2.32	3.48 ± 2.89 ^b^	5.14 ± 2.29	**0.020 ^s^**
*p* value (*)	**0.664**	**0.026 ^s^**	**0.508**	
***P. intermedia* (log10)**				
Baseline	2.96 ± 3.00	2.19 ± 2.97	2.33 ± 2.88	0.481
3 m	3.81 ± 3.15 ^a^	1.87 ± 2.66	2.11 ± 2.76	**0.011 ^s^**
6 m	3.88 ± 2.93	1.48 ± 2.51	2.19 ± 2.95	**0.011 ^s^**
*p* value (*)	**0.076**	**0.538**	**0.360**	
***F. nucleatum* (log10)**				
Baseline	6.87 ± 0.98	6.53 ± 1.91	6.49 ± 1.28	0.455
3 m	6.87 ± 1.13	6.42 ± 1.59	6.74 ± 1.11	0.456
6 m	6.71 ± 1.03	6.51 ± 1.03	6.99 ± 0.95 ^b^	0.194
*p* value (*)	**0.432**	**0.764**	**0.012 ^s^**	
***C. rectus* (log10)**				
Baseline	4.35 ± 2.78	4.15 ± 3.02	3.66 ± 3.02	0.534
3 m	4.21 ± 3.09	2.96 ± 2.97 ^a^	3.90 ± 2.85	0.129
6 m	3.99 ± 3.07 ^c^	2.38 ± 3.14 ^b^	4.48 ± 3.02	0.056
*p* value (*)	**0.202**	**0.055**	**0.404**	
***F. allocis* (log10)**				
Baseline	5.23 ± 2.38	4.98 ± 2.60	4.92 ± 2.74	0.959
3 m	4.93 ± 2.75	3.88 ± 2.59 ^a^	5.21 ± 2.34	**0.025 ^s^**
6 m	4.99 ± 2.72	3.31 ± 3.13 ^b^	5.37 ± 2.55	**0.016 ^s^**
*p* value (*)	**0.180**	**0.016 ^s^**	**0.318**	

(£) Kruskal–Wallis test. (*) Friedman test. ^a^: statistically significantly different between baseline and 3 months (*p* < 0.05). ^b^: statistically significantly different between baseline and 6 months (*p* < 0.05). ^c^: statistically significantly different between 3 and 6 months (*p* < 0.05). ^s^: statistically significant (*p* < 0.05).

## Data Availability

Data is available per request at Raluca.cosgarea@gmail.com.
